# Supplementary material for: A Novel Missense Mutation in ERCC8 Co-Segregates with Cerebellar Ataxia in a Consanguineous Pakistani Family
Source: Cells. 2022 Sep 30;11(19):3090. doi: 10.3390/cells11193090 (PMC9564319; doi:10.3390/cells11193090)
Supplement: Supplementary file 1 [file cells-1934066-supplementary.pdf › cells-1934066-supplementary.pdf]

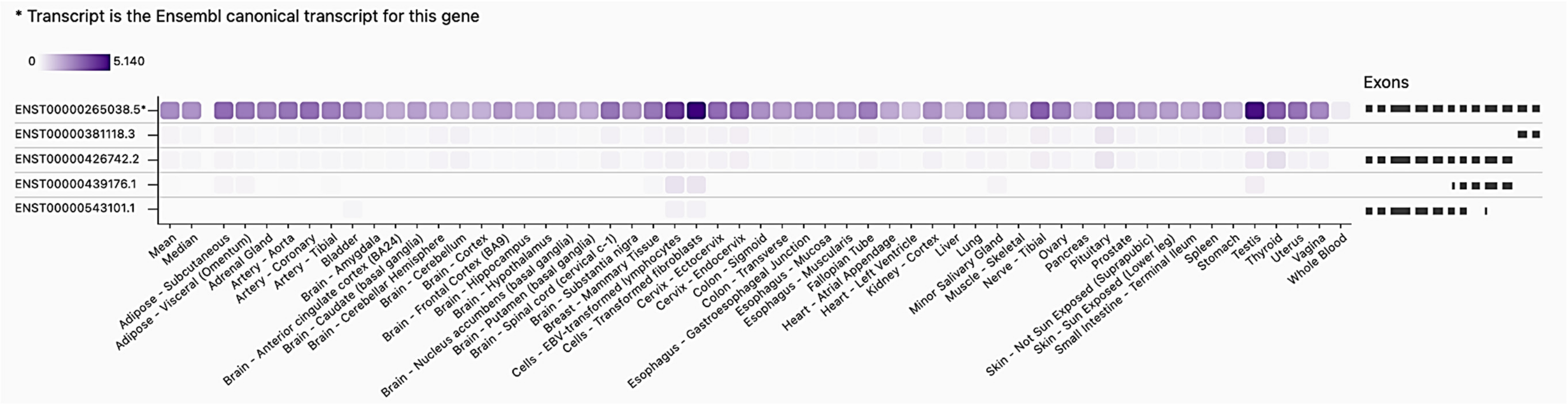

Figure S1. *ERCC8* transcript isoforms expression in different tissues as shown by GTEx.
